# Serum YKL-40 as a biomarker for liver fibrosis in chronic hepatitis B patients with normal and mildly elevated ALT

**DOI:** 10.1007/s15010-018-1136-2

**Published:** 2018-03-29

**Authors:** Linlin Yan, Yongqiong Deng, Jiyuan Zhou, Hong Zhao, Guiqiang Wang, China HepB-Related Fibrosis Assessment Research Group, Da-Zhi Zhang, Shi-Bin Xie, Xu-Qing Zhang, Qing Xie, Jun Cheng, Weifeng Liang, Fanping Meng, Lang Bai, Jun Li

**Affiliations:** 1 Department of Infectious Disease, Center for Liver Disease, Peking University First Hospital, No. 8, Xishiku Street, Xicheng District, Beijing, 100034 China; 2 The Department of Dermatology, The Affiliated Hospital of Southwest Medical University, Luzhou, 646000 Sichuan China; 3 The Collaborative Innovation Center for Diagnosis and Treatment of Infectious Diseases, Zhejiang University, Hangzhou, Zhejiang China; 4 Peking University International Hospital, Beijing, China

**Keywords:** YKL-40, Chronic hepatitis B, Liver fibrosis

## Abstract

**Purpose:**

YKL-40 is a chitinase-like protein expressed in multiple tissues including liver and is reported as a fibrosis marker. This study aimed to determine whether YKL-40 could serve as a diagnostic marker for the assessment of liver fibrosis in chronic hepatitis B patients with normal and mildly elevated ALT.

**Methods:**

Six hundred and eighty-five patients with chronic hepatitis B infection were enrolled in this study from October 2013 to March 2016. All patients underwent liver biopsy and then staged based on Ishak histological system. Serum YKL-40 levels were measured by Human Magnetic Luminex Assays.

**Results:**

Among chronic hepatitis B patients with normal and mildly elevated ALT, almost more than 30% of patients have significant liver fibrosis. Serum YKL-40 levels increased significantly in parallel with the progression of fibrosis in patients with ALT less than two times the upper limit of normal range (*P *< 0.0001). Multivariate analysis revealed that serum YKL-40, hyaluronic acid, PLT, and AST were independently associated with significant fibrosis. We established a novel YKL-40-based fibrosis model for patients with ALT less than two times the upper limit of normal range (ULN). YKL-40 model was superior to APRI, FIB-4, Forns’ index, and Hui model for diagnosis of significant fibrosis in patients with ALT < 2ULN, with AUROCs of 0.786 [95% confidence interval (CI) 0.726–0.846] in the training group, 0.831 (95%CI 0.752–0.910) in the validation group and 0.801 (95%CI 0.753–0.849) in the entire cohort.

**Conclusion:**

Serum YKL-40 is a feasible biomarker of liver fibrosis in chronic hepatitis B patients. YKL-40 model was superior to APRI, FIB-4, Forns’ index and Hui model for diagnosis of significant fibrosis in patients with normal and mildly elevated ALT.

**Electronic supplementary material:**

The online version of this article (10.1007/s15010-018-1136-2) contains supplementary material, which is available to authorized users.

## Introduction

Chronic hepatitis B (CHB) infection remains a major global health burden; approximately, 350–400 million individuals were infected [1]. The burden of chronic hepatitis B infection is serious in China, with an estimated 120 million people infected, and 0.3 million deaths annually [2]. In China, a proportion of CHB patients are asymptomatic accompanied by normal and mildly elevated alanine transaminase (ALT, ALT levels are less than two times the upper limit of normal). The guidelines of American Association for the Study of Liver Diseases (AASLD) and Asian Pacific Association for the Study of the Liver for the management of CHB recommend antiviral treatment when ALT levels are two times the upper limit of normal (ULN), and monitoring or performing liver biopsy (especially for patients > 40 years) to assess if significant histologic disease is present when ALT levels are less than two times the upper limit of normal (ULN) [3, 4]. However, CHB patients with normal and mildly elevated ALT levels may not have healthy livers. Several studies indicated that moderate inflammation and/or advanced fibrosis was present in 28–37% of CHB patients who had persistently normal ALT [5–7]. These studies suggested that CHB patients with normal ALT might have histologically significant disease, an indication for antiviral treatment. Use of ALT without resorting to liver biopsy may miss a certain proportion of patients with histologically significant disease who may benefit from antiviral therapy. Liver biopsy remains the gold standard for assessing liver fibrosis in CHB patients. However, liver biopsy has several limitations including invasiveness, risk of complications, sampling error, and cost [8], which limited its application in assessing and dynamic monitoring of liver fibrosis. Currently, multiple noninvasive methods based on laboratory tests have been developed as surrogates to assess liver fibrosis, such as aspartate aminotransferase–platelet index (APRI), fibrosis index based on the four factors (FIB-4), Forns’ index [9], and Hui model [10]. Gao et al. [11] had reported a noninvasive model, consisting of aspartate transaminase (AST), HBsAg, platelet, and albumin, to predict significant liver histology change [necroinflammatory activity grade (*G*) ≥ 2 or fibrosis stage (*S*) ≥ 2] in HBeAg-positive CHB with ALT ≤ 2ULN. Gao’s model had an area under the receiver operating characteristic curve of 0.868, which was significantly higher than APRI and FIB-4. However, there is no noninvasive method to predict significant fibrosis in CHB patients with ALT < 2ULN regardless of HBeAg status and HBV DNA levels.

YKL-40 (chitinase-3-like-1, or human cartilage glycoprotein-39) is a member of the mammalian chitinase family [12] and is secreted by a variety of cells, including neutrophils, macrophages, and vascular smooth muscle cells [13]. YKL-40 is thought to be involved in remodeling of the extracellular matrix and in inflammatory processes [14]. YKL-40, as the growth factor for fibroblasts and chemoattractant for endothelial cells, is also believed to modulate angiogenesis during tissue damage [15, 16]. Recently, YKL-40 mRNA expression was found in human liver [17], and serum YKL-40 levels were associated with liver fibrosis in patients with chronic liver disease [18]. Immunohistochemical studies have shown that YKL-40 is expressed in fibrotic areas of the liver [17, 19]. Based on these supporting evidences, serum YKL-40 has been evaluated as a noninvasive marker of fibrotic liver diseases, including alcoholic liver disease [20], non-alcoholic fatty liver disease [21] and chronic hepatitis C-induced liver fibrosis [22, 23]. Therefore, we recently proposed a hypothesis that serum YKL-40 may be a potential biomarker for differentiating significant fibrosis in chronic hepatitis B patients with normal and mildly elevated ALT.

In this study, we identified the proportion of significant fibrosis in CHB patients with normal and mildly elevated ALT. We measured the serum levels of YKL-40 and compared them with fibrosis stages to evaluate the feasibility of YKL-40 as a biomarker of liver fibrosis in patients with normal and mildly elevated ALT levels.

## Patients and methods

### Patients

A total of 685 patients with chronic HBV infection from 24 hospitals located in mainland China were enrolled in this study between October 2013 and March 2016. Of which, 460 patients have ALT levels less than two times the upper limit of normal range (ULN), and they were randomly divided into a training group (*n* = 307) and a validation group (*n* = 153). They all underwent liver biopsies. Inclusion and exclusion criteria were described previously [24]. All patients provided written informed consent for research use of their clinical data and specimens. This study was approved by the Ethics Committee of Peking University First Hospital. The detailed protocol for the clinical trial was registered at clinicaltrials.gov (NCT01962155) and chictr.org (ChiCTR-DDT-13003724).

### Histological staging

Ultrasonography-guided liver biopsies with a minimal length of 20 mm (at least 11 portal tracts) were routinely performed at each hospital according to a standardized protocol after receiving the patient’s written informed consent. Pathological interpretations were conducted in the Department of Pathology at You An Hospital affiliated to the Capital Medical University. The histopathological examination rules were previously reported [24]. Fibrosis stages were assessed according to Ishak criteria [25]. Significant fibrosis was defined as F3.

### Examination of serum markers

The biochemical and hematological parameters were routinely detected by standard methods in local hospitals. Serum HBV DNA (range 2.0 × 10^1^–1.7 × 10^8^ IU/ml) was measured by the COBAS AmpliPrep/COBAS TaqMan (Roche Diagnostics, Basel, Switzerland). Serum HBsAg (range of 20–52,000 IU/ml) was quantified using the Roche Elecsys HBsAg II assay (Roche Diagnostics, Penzberg, Germany). The serum levels of YKL-40 were determined using Human Magnetic Luminex® Assays (LXSAHM-08, R&D Systems, Inc, Minneapolis, MN, USA) according to the manufacturer’s instructions. The serum concentrations of hyaluronic acid (range of 2–200 μg/L), laminin (5–900 μg/L), were measured using a chemiluminescence immunoassay kit (Yuande Bio-Medical Engineering Co., Ltd, Beijing, China).

### Noninvasive fibrosis scores

Noninvasive assessment of fibrosis, APRI, and FIB4 was calculated according to the following formulae: APRI = [(AST/ULN)/platelet(× 10^9^/L)] × 100; FIB4 = (age × AST)/[platelet(× 10^9^/L) × ALT^1/2^]. Forns’ index [9] and Hui model [10] were obtained from reported research.

### Statistical analysis

Quantitative variables were expressed as mean ± standard deviation (SD) and categorical variables were expressed as proportions. For normally and non-normally distributed variables, the differences between the groups were analyzed using Student *t* test and Mann–Whitney *U* test, respectively. For categorical variables, Chi-square test was used to compare the differences in proportions. Spearman’s rank test was used to analyze the correlations between different variables and fibrosis stages. We performed multivariate backward logistic regression analysis to determine the independent variables of significant fibrosis. Receiver operating characteristic curve (ROC) was used to assess the performance of noninvasive models for staging significant fibrosis. The diagnostic performance of different variables was evaluated based on the area under the receiver operating characteristic curve (AUROC). SPSS 16.0 software (SPSS, Inc., Chicago, IL, USA) was used for statistical analyses. *P* < 0.05 were considered statistically significant.

## Results

### Patient’s characteristics

A total of 685 patients were enrolled in this study; seven patients were excluded because of unqualified liver tissue. The remaining 678 patients with chronic HBV infection were analyzed, of which 460 patients with ALT less than two times the upper limit of normal range (ULN). The baseline characteristics of the study patients are shown in Table 1. There were no significant distributional differences in fibrosis stages between the group of patients with ALT ≥ 2 × ULN and the group of patients with ALT < 2 × ULN (*P *= 0.312, Table 1). This result indicated the presence of significant or more severe fibrosis in patients with ALT < 2 × ULN, patients who do not meet the treatment criteria recommended by AASLD guideline.

**Table 1:** Patients’ characteristics

	ALT ≥ 2 × ULN (*n* = 218)	ALT < 2 × ULN (*n* = 460)	*P* value
Age (median, ≥ 40 years %)	36, 77 (35.3%)	38, 205 (44.6%)	0.024
Gender (male %)	184 (84.4%)	345 (75.0%)	0.006
BMI (median, ≥ 24 kg/m^2^ %)	23.3, 76 (34.9%)	23.0, 165 (35.9%)	0.864
HBsAg (log_10_IU/mL)	3.59 ± 0.77	3.56 ± 0.88	0.409
AST (U/L)	116.55 ± 109.99	35.50 ± 17.84	< 0.001
ALP (U/L)	91.64 ± 29.93	77.31 ± 26.07	< 0.001
GGT (U/L)	82.62 ± 69.51	41.50 ± 47.42	< 0.001
Albumin (g/L)	43.67 ± 5.85	44.48 ± 5.26	0.002
TBil (µmol/L)	18.30 ± 15.33	16.94 ± 22.78	0.017
PT (s)	12.94 ± 1.51	12.56 ± 1.49	0.001
PLT (× 10^9^/L)	170.64 ± 52.76	172.33 ± 59.08	0.635
Hyaluronic acid (ug/L)	149.05 ± 102.82	115.26 ± 71.14	< 0.001
Laminin (ug/L)	179.54 ± 302.39	84.24 ± 177.79	< 0.001
PIIINP (ug/L)	5.65 ± 11.42	3.65 ± 5.04	< 0.001
Collagen IV (pg/mL)	1120.00 ± 628.19	896.98 ± 540.96	< 0.001
YKL-40 (log_10_ pg/mL)	4.46 ± 0.38	4.47 ± 0.38	0.718
sCD163(log_10_ pg/mL)	6.20 ± 0.36	6.01 ± 0.33	< 0.001
MMP-1 (log_10_ pg/mL)	3.47 ± 0.31	3.48 ± 0.32	0.566
MMP-2 (log_10_ pg/mL)	5.28 ± 0.10	5.26 ± 0.10	0.058
MMP-3 (log_10_ pg/mL)	4.17 ± 0.25	4.17 ± 0.26	0.624
MMP-9 (log_10_ pg/mL)	4.85 ± 0.41	4.87 ± 0.45	0.770
TIMP-1 (log_10_ pg/mL)	5.08 ± 0.12	5.06 ± 0.13	0.018
HBeAg status/HBV DNA(IU/mL) (*n* %)			0.020
G1 e + , HBV DNA ≥ 2 × 10^7^	86 (39.4%)	128(27.6)	
G2 e + , 20,000 ≤ HBV DNA < 2 × 10^7^	54 (24.8)	116 (25.4)	
G3 e + , HBV DNA < 20,000	9 (4.1)	25 (5.7)	
G4 e − , HBV DNA ≥ 2000	59 (27.1)	152 (32.8)	
G5 e − , HBV DNA < 2000	10 (4.6)	39 (8.5)	
Fibrosis stages (*n* %)			0.312
*F*0–2	128 (58.7%)	291 (63.2%)	
*F*3	48 (22.0%)	85 (18.5%)	
*F*4	33 (15.1%)	68 (14.8%)	
*F*5–6	9 (4.1%)	16 (3.5%)	

Data presented as mean ± SD or no. (%)

*BMI* body mass index, *HBsAg* hepatitis B surface antigen, *AST* aspartate transaminase, *ALP* alkaline phosphatase, *GGT* gamma-glutamyl transpeptidase, *TBil* total bilirubin, *PT* prothrombin time, *PLT* platelet counts, *PIIINP* N-terminal peptide of type III procollagen, *YKL*-*40* chitinase 3-like-1, *sCD163* soluble CD163, *MMP* matrix metalloproteinase, *TIMP*-*1* tissue inhibitor of metalloproteinase 1, *HBeAg* hepatitis B e antigen, *HBV* hepatitis B virus, *ULN* upper limit of normal

### ALT was not a perfect surrogate marker for liver histology

**Fig. 1:**
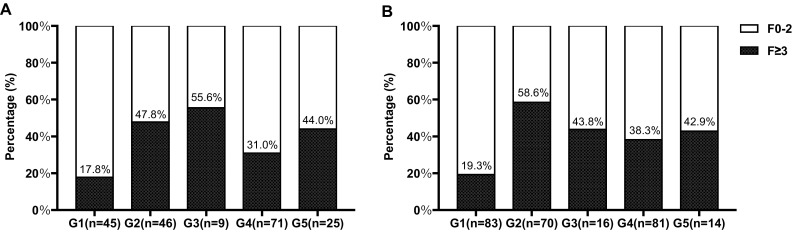
Proportion of patients with significant fibrosis in the group of G1–G5 in chronic hepatitis B patients. Patients with **a** normal ALT and **b** mildly elevated ALT

For patients with ALT < 2 × ULN, they were stratified (from G1 to G5) according to the status of HBeAg and the levels of HBV DNA, as shown in Table 1. In patients with normal ALT, differences in the proportion of significant fibrosis were statistically significant (*P *= 0.015, Fig. 1a). Overall, more than 30% of patients had significant fibrosis, besides G1 (immuno-tolerant phase) with 17.8% incidence of significant fibrosis. Similar results were obtained in patients with mildly elevated ALT (*P *<0.0001, Fig. 1b). Regarding the incidence of significant fibrosis between patients with normal ALT and patients with mildly elevated ALT, there were no significant differences (data not shown). This result suggested that ALT levels and fibrosis are not always consistent in CHB patients.

### Serum YKL-40 levels increased with the progression of fibrosis

**Fig. 2:**
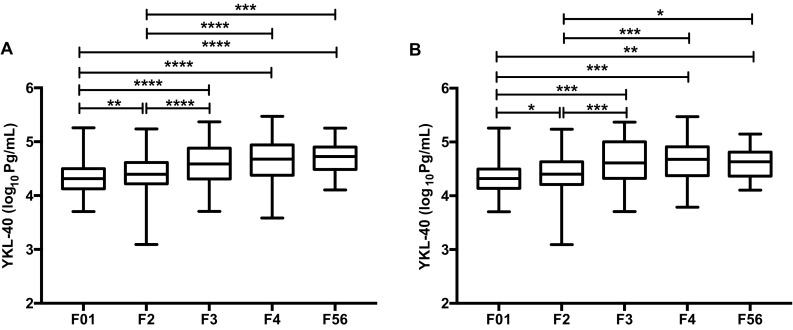
Associations between serum YKL-40 levels and liver fibrosis. **a** YKL-40 in total patients, **b** YKL-40 in patients with ALT < 2 × ULN. *P *< 0.0001 for all fibrosis stags. ****P < 0.0001, ***P < 0.001, **P < 0.01, *P < 0.05

Serum YKL-40 levels were measured to assess the feasibility of YKL-40 as a biomarker of fibrosis in CHB patients. Serum levels of YKL-40 throughout different fibrosis stages are shown in Fig. 2. In the total patients, serum YKL-40 levels increased in parallel with the progression of fibrosis, showing significant difference between fibrosis stages (F01 vs F2–F56, F2 vs F3–F56) (*P *< 0.0001, Fig. 2a). In patients with ALT < 2 × ULN, similar results were obtained as in the total patients (Fig. 2b). In addition, serum YKL-40 levels were positively correlated with hyaluronic acid, laminin, PIIINP, Collagen, and AST, while they were negatively correlated with platelet count (Supplementary Table 1).

### Development of YKL-40-based fibrosis model in patients with ALT < 2 × ULN

To determine the ability of YKL-40 to diagnose significant fibrosis, all CHB patients with ALT < 2 × ULN were divided into a training group and a validation group. There was no statistical difference between training group and validation group about any parameters (Supplementary Table 2). In the training group, univariate analysis found that serum YKL-40, hyaluronic acid, laminin, PIIINP, Collagen IV, sCD163, and MMP-2 were positively associated with significant fibrosis (Table 2). PLT was inversely associated with significant fibrosis (Table 2). Multivariate analysis revealed that YKL-40 [odd ratio (OR) 2.330, 95% confidence interval (CI) 1.019–5.330, *P *= 0.045], hyaluronic acid (HA), PLT, and AST were independent factors of significant fibrosis (Table 3). We performed backward logistic regression analysis and established a novel YKL-40 based model for CHB patients with ALT < 2 ULN:

**Table 2:** Univariate analysis of clinical parameters and biomarkers with significant fibrosis in the training group (*n* = 307)

	*F*0–2 (*n* = 194)	*F* ≥ 3 (*n* = 113)	*P* value
Age (≥ 40 years %)	37.54 ± 10.12	42.54 ± 10.92	< 0.0001
Gender (male %)	141 (72.68%)	83 (73.45%)	> 0.9999
BMI (≥ 24 kg/m^2^ %)	22.88 ± 2.95	23.50 ± 2.74	0.096
HBsAg (log_10_IU/mL)	3.71 ± 0.88	3.28 ± 0.72	< 0.0001
ALT (U/L)	42.16 ± 17.61	44.55 ± 16.04	0.258
AST (U/L)	32.52 ± 15.72	42.36 ± 21.75	< 0.0001
ALP (U/L)	72.61 ± 19.21	85.90 ± 33.97	0.002
GGT (U/L)	35.60 ± 45.56	54.91 ± 47.21	< 0.0001
Albumin (g/L)	45.06 ± 4.53	43.58 ± 6.78	0.003
TBil (µmol/L)	15.45 ± 17.01	20.84 ± 38.75	0.002
PT (s)	12.32 ± 1.27	12.85 ± 1.43	0.002
PLT (× 10^9^/L)	187.06 ± 49.56	145.23 ± 53.21	< 0.0001
Hyaluronic acid (ug/L)	93.89 ± 41.27	151.04 ± 96.27	< 0.0001
Laminin (ug/L)	48.21 ± 91.38	126.52 ± 204.21	< 0.0001
PIIINP (ug/L)	3.12 ± 6.14	4.53 ± 4.56	< 0.0001
Collagen IV (pg/mL)	782.48 ± 387.26	1037.84 ± 584.14	< 0.0001
YKL-40 (log10 pg/mL)	4.39 ± 0.35	4.62 ± 0.40	< 0.0001
SCD163 (log10 pg/mL)	5.94 ± 0.32	6.12 ± 0.32	< 0.0001
MMP-1 (log10 pg/mL)	3.49 ± 0.32	3.46 ± 0.31	0.501
MMP-2 (log10 pg/mL)	5.24 ± 0.10	5.28 ± 0.10	0.001
MMP-3 (log10 pg/mL)	4.17 ± 0.27	4.18 ± 0.26	0.740
MMP-9 (log10 pg/mL)	4.86 ± 0.46	4.86 ± 0.40	0.752
TIMP-1 (log10 pg/mL)	5.05 ± 0.13	5.08 ± 0.13	0.134

*BMI* body mass index, *HBsAg* hepatitis B surface antigen, *ALT* alanine transaminase, *AST* aspartate transaminase, *ALP* alkaline phosphatase, *GGT* gamma-glutamyl transpeptidase, *TBil* total bilirubin, *PT* prothrombin time, *PLT* platelet counts, *PIIINP* N-terminal peptide of type III procollagen, *YKL*-*40* chitinase 3-like-1, *sCD163* soluble CD163, *MMP* matrix metalloproteinase, *TIMP*-*1* tissue inhibitor of metalloproteinase 1

**Table 3:** Multivariate logistic regression analysis of independent predictors for significant fibrosis in the training group (*n* = 307)

	Coefficient	OR	95%CI	*P* value
AST (U/L)	0.032	1.033	1.009–1.057	0.007
PLT (× 10^9^/L)	− 0.012	0.988	0.982–0.995	< 0.0001
Hyaluronic acid (ug/L)	0.012	1.013	1.005–1.020	0.001
YKL-40 (log10 pg/mL)	0.846	2.330	1.019–5.330	0.045
Constant	− 4.758	0.009	–	0.018

YKL-40 model = 0.032 × AST − 0.012 × PLT + 0.012 × HA + 0.846 × log10 (YKL-40) − 4.752

YKL-40 model = 0.032 × AST − 0.012 × PLT + 0.012 × HA + 0.846 × log10 (YKL-40) − 4.752.

### Diagnostic performance of YKL-40 model for significant fibrosis

**Fig. 3:**
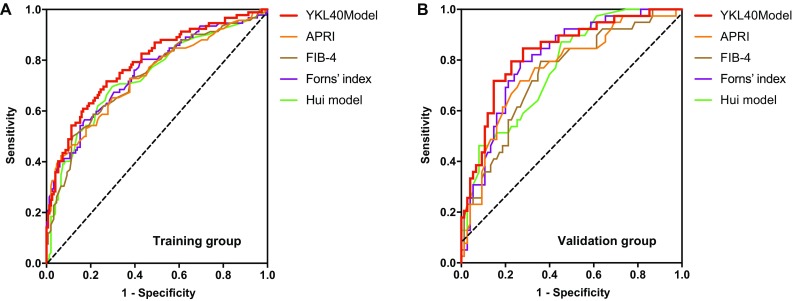
Receiver operating characteristic curve (ROC) analysis showing the diagnostic performance of noninvasive models for significant fibrosis. Area under the ROC curves (AUROCs) of YKL-40 model, ARPI, FIB4, Forns’ index and Hui model in the diagnosis of significant fibrosis in CHB patients with ALT < 2ULN. **a** Training group and **b** validation group

YKL-40 model had an area of 0.786 (95%CI 0.726–0.846) under the ROC curve in predicting significant fibrosis in the training group, with 71.74% sensitivity, 72.85% specificity, 61.68% PPV, and 80.88% NPV at the cut-off point of − 0.56. It was superior to that of APRI [0.736 (95%CI 0.670–0.803)], FIB-4 [0.735 (95%CI 0.669–0.801)], Forns’ index [0.753 (95%CI 0.688–0.817)], and Hui model [0.734 (95%CI 0.667–0.801)] (Fig. 3a, Table 4). The area under the ROC curve of YKL-40 model in the validation group was 0.831 (95%CI 0.752–0.910), with 71.79% sensitivity, 85.33% specificity, 71.79% PPV, and 85.33% NPV at the cut-off point of − 0.33, which was also higher than that of APRI, FIB-4, Forns’ index, and Hui model (Fig. 3b, Table 4). In the entire cohort, YKL-40 model had an area of 0.801 (95%CI 0.753–0.849) under the ROC curve in predicting significant fibrosis (data not shown**)**.

**Table 4:** Receiver operating characteristics curve (ROC) analysis of noninvasive models for the diagnosis of significant fibrosis in CHB patients with ALT < 2ULN

	AUROC (95%CI)	Cut-off value	Sensitivity (%)	Specificity (%)	PPV (%)	NPV (%)
Training group						
YKL-40 model	0.786 (0.726–0.846)	− 0.56	71.74	72.85	61.68	80.88
APRI	0.736 (0.670–0.803)	0.76	42.39	93.38	79.60	72.68
FIB-4	0.735 (0.669–0.801)	1.33	50.00	88.08	71.88	74.30
Forns’ index	0.753 (0.688–0.817)	7.75	56.52	82.78	66.66	75.76
Hui model	0.734 (0.667–0.801)	0.12	69.57	70.20	58.72	79.11
Validation group						
YKL-40 model	0.831 (0.752–0.910)	− 0.33	71.79	85.33	71.79	85.33
APRI	0.762 (0.668–0.855)	0.50	71.79	73.33	58.33	83.33
FIB-4	0.743 (0.648–0.838)	1.14	79.49	64.00	53.45	85.72
Forns’ index	0.804 (0.723–0.886)	7.12	79.49	73.33	60.78	87.30
Hui model	0.771 (0.684–0.857)	0.08	87.18	54.67	50.00	89.13

## Discussion

Serum ALT is commonly used to assess liver histology activity and to guide antiviral therapy in patients with liver disease. However, results of the present study showed that, ALT levels and fibrosis are not always consistent in CHB patients. We observed that a high proportion (> 30%) of CHB patients with normal and mildly elevated (1-2ULN) ALT have significant fibrosis regardless of the state of HBeAg and the levels of HBV DNA (Fig. 1, G2–G5). Even for patients in the immunO-tolerant phase (Fig. 1, G1), 17.8 and 19.3%, respectively, have significant fibrosis. Our present findings are consistent with the previous reports that patients with chronic HBV infection can display normal and mildly elevated ALT levels despite significant histological injury [6, 7, 26]. A meta-analysis [26] concluded that approximately one-fifth of CHB patients with ALT ≤ 40 IU/L may have significant hepatic fibrosis. Lai et al. [7] found that 37% of CHB patients with persistently normal ALT had significant fibrosis and inflammation. According to current guidelines, antiviral therapy should be initiated immediately for patients with significant fibrosis [3, 4]. Our results confirmed that ALT was not a perfect surrogate marker for liver histology, because ALT failed to identify many patients who might benefit from antiviral therapy. The “gray zone” patients were defined as those patients with normal and mildly elevated ALT. Because of the high proportion of significant liver disease in the “gray zone” patients, it is highly important to assess liver fibrosis. Liver biopsy, a gold standard for assessing liver fibrosis, is not suitable for regular applications due to the limitations of invasive, finite, complications, and cost [8]. Noninvasive models such as APRI and FIB-4 using biochemical laboratory index have been proposed to replace liver biopsy to assess liver fibrosis. Therefore, it is reasonable to evaluate “gray zone” patients based on such noninvasive methods, and then to decide whether initiating antiviral treatment or not.

Within the present study, we assessed the relationship between serum markers, including YKL-40, hyaluronic acid, laminin, PIIINP, Collagen IV, sCD163 and metalloproteinases, and liver fibrosis in patients with normal and mildly elevated ALT. Of note, our results indicated that serum YKl-40 levels significantly correlated with fibrosis stages as assessed by Ishak score. Serum levels of YKL-40 also increased in non-alcoholic fatty liver disease (NAFLD) and chronic hepatitis C-induced liver fibrosis [21, 22, 27, 28]. It has been reported that YKL-40 is a growth factor for fibroblasts and is expressed in active liver fibrotic areas [29, 30]. In addition, the progression of fibrosis rate per year linearly correlates with the serum levels of YKL-40 [23]. These observations further strengthen the possibility that YKL-40 is involved in hepatic fibrogenesis in patients with HBV infection and is a useful biomarker for hepatic fibrosis. It is critical to discriminate patients with significant fibrosis, a stage which represent an indication for antiviral therapy, from the “gray zone” patients. Our univariate analysis revealed that serum YKL-40, hyaluronic acid, laminin, PIIINP, Collagen IV, sCD163, and MMP-2 were associated with significant fibrosis. However, multivariate analysis showed that only YKL-40, hyaluronic acid and two laboratory parameters, PLT and AST, retained significance when combined with other clinical parameters. Series studies have demonstrated that combination of multiple serum markers could improve the sensitive, specific, and reproducible [31, 32]. Based on our findings, a four-variable model including two serum fibrosis markers (log_10_YKL-40, hyaluronic acid) and two routinely laboratory tests (PLT, AST) was derived via backward logistic regression analysis to detect significant fibrosis. Hyaluronic acid is synthesized by stellate cells and is involved in fibrogenesis; it has been identified as one of the serum markers of liver fibrosis in non-alcoholic steatohepatitis (NASH) and chronic hepatitis C [31–34]. Regarding PLT, our finding is consistent with the previous studies that found decreased platelet counts are associated with more severe hepatic fibrosis [35, 36].

Identification of patients, who actually had significant hepatic fibrosis, diagnosed as “none treatment required” according to ALT levels, is very important. Significant fibrosis is an important endpoint of clinical antiviral therapy [37, 38]. The aim of this study was to develop an accurate noninvasive fibrosis model applied to “gray zone” CHB patients. Over the past 20 years, various noninvasive fibrosis models have emerged. The most widely used two scores, APRI and FIB-4, and Forns’ index, are based on patients with hepatitis C infection [39]. APRI and FIB-4 have been validated and recommended for evaluation of liver fibrosis in CHB patients [39, 40]. Hui model is based on patients with HBV [10] while lacking of clinical validation (Supplementary Table 3). Furthermore, the diagnostic performances of the above models for fibrosis assessment in CHB patients with normal and mildly elevated ALT have not been validated in large cohorts. A recent analysis of APRI and FIB-4 in 231 HBV-infected patients with normal and mildly elevated ALT founded limited diagnostic value for significant fibrosis [41]. In this study, we developed a YKL-40 model in 460 CHB patients with normal and mildly elevated ALT, and then, we compared the performances of the five noninvasive models to diagnose significant fibrosis. For the identification of patients with significant fibrosis, the AUROCs for patients with ALT < 2ULN were 0.736 for APRI and 0.735 for FIB-4 in the training group, compared with 0.762 for APRI and 0.743 for FIB-4 in the validation group, showing similar performance as previous reported [41]. We found that YKL-40 model produced the best performances compared to existing scores, with AUROCs of 0.786 in the training group, 0.831 in the validation group and 0.801 in the entire cohort in predicting significant fibrosis for patients with ALT < 2ULN.

These findings indicated that combined measurement of serum YKL-40, hyaluronic acid, PLT and AST, via YKL-40 model can help identify “gray zone” CHB patients with significant fibrosis who should be treated immediately.

The limitation of this study is that the performance of YKL-40 model has not been validated by longitudinal data and future prospective studies should be performed. In addition, the mechanisms of YKL-40 in liver fibrogenesis of chronic HBV infection have not been clarified and this will require the basic research works.

In conclusion, the present study supports a fact that in China, significant liver fibrosis is present in a high proportion of CHB patients with normal and mildly elevated ALT levels regardless of HBeAg status and HBV DNA levels. In CHB patients with ALT < 2ULN, serum YKL-40 levels were independently associated with significant fibrosis and could be a feasible biomarker reflecting liver fibrosis. YKL-40 model was superior to existing scores in diagnosing significant fibrosis in CHB patients with normal and mildly elevated ALT. This finding offered a promising method to identify those “gray zone” patients who may benefit from antiviral therapy.

## Electronic supplementary material

Below is the link to the electronic supplementary material.

Supplementary Table 1. Correlations between serum YKL-40 levels and routine parameters and biomarkers in patients with ALT<2ULN (n=460).

Supplementary Table 2. Characteristics of patients in training group and validation group

Supplementary Table 3. Comparisons of the five noninvasive fibrosis models.

Supplementary material 1 (DOCX 76 kb)

## Abbreviations

HBV: Hepatitis B virus
CHB: Chronic hepatitis B
HBsAg: Hepatitis B surface antigen
HBeAg: Hepatitis B e antigen
BMI: Body mass index
ALT: Alanine transaminase
AST: Aspartate transaminase
ALP: Alkaline phosphatase
GGT: Gamma-glutamyl transpeptidase
TBil: Total bilirubin
PT: Prothrombin time
PLT: Platelet counts
HA: Hyaluronic acid
LN: Laminin
PIIINP: N-terminal peptide of type III procollagen
YKL-40: Chitinase 3-like-1
sCD163: Soluble CD163
MMP: Matrix metalloproteinase
TIMP-1: Tissue inhibitor of metalloproteinase 1
ULN: Upper limit of normal
ROC: Receiver operating characteristic curve
AUROC: Area under the receiver operating characteristic curve
SD: Standard deviation
CI: Confidence interval
