# Supplementary material for: Serum YKL-40 as a biomarker for liver fibrosis in chronic hepatitis B patients with normal and mildly elevated ALT
Source: Infection. 2018 Mar 29;46(3):385–93. doi: 10.1007/s15010-018-1136-2 (PMC5976691; doi:10.1007/s15010-018-1136-2)
Supplement: Supplementary file 1 — Supplementary material 1 (DOCX 76 kb) [file 15010_2018_1136_MOESM1_ESM.docx]

**Supplementary Material**

**Supplementary Table 1.** Correlations between serum YKL-40 levels and routine parameters and biomarkers in patients with ALT<2ULN (n=460).

|  | **Total (n=678)** | |  | **ALT<2ULN (n=460)** | |
| --- | --- | --- | --- | --- | --- |
|  | **Spearman’s rho** | **P-Value** |  | **Spearman’s rho** | **P-Value** |
| ALT (U/L) | 0.041 | 0.291 |  | 0.062 | 0.183 |
| AST (U/L) | 0.153 | <0.0001 |  | 0.240 | <0.0001 |
| PLT (×10^9^/L) | -0.195 | <0.0001 |  | -0.226 | <0.0001 |
| Hyaluronic (ug/L) | 0.369 | <0.0001 |  | 0.377 | <0.0001 |
| Laminin (ug/L) | 0.226 | <0.0001 |  | 0.218 | <0.0001 |
| PIIINP (ug/L) | 0.233 | <0.0001 |  | 0.225 | <0.0001 |
| Collagen IV (Pg/ML) | 0.333 | <0.0001 |  | 0.337 | <0.0001 |
| MMP-1 (log_10_ Pg/ML) | -0.097 | 0.012 |  | -0.113 | 0.015 |
| MMP-2 (log_10_ Pg/ML) | 0.209 | <0.0001 |  | 0.184 | <0.0001 |
| MMP-3 (log_10_ Pg/ML) | -0.003 | 0.947 |  | -0.044 | 0.347 |
| MMP-9 (log_10_ Pg/ML) | -0.064 | 0.098 |  | -0.045 | 0.335 |
| TIMP-1 (log_10_ Pg/ML) | 0.005 | 0.892 |  | 0.023 | 0.622 |

*ALT* alanine transaminase, *AST* aspartate transaminase, *PLT* platelet counts, *PIIINP* N-terminal peptide of type III procollagen, *MMP* matrix metalloproteinase, *TIMP-1* tissue inhibitor of metalloproteinase 1, *ULN* upper limit of normal.

**Supplementary Table 2.** Characteristics of patients in training group and validation group

|  | **Training Group**  **(n=307)** | **Validation Group (n=153)** | ***P* value** |
| --- | --- | --- | --- |
| Age (median, year) | 39.38±10.68 | 38.57±10.39 | 0.495 |
| Gender (male,%) | 224 (72.96%) | 121 (79.08%) | 0.171 |
| BMI (median, kg/m^2^) | 23.11±2.89 | 23.41±2.59 | 0.277 |
| HBsAg (log_10_IU/mL) | 3.55±0.85 | 3.59±0.94 | 0.185 |
| ALT (U/L) | 43.04±17.06 | 43.12±17.23 | 0.997 |
| AST (U/L) | 36.12±18.74 | 34.26±15.88 | 0.117 |
| ALP (U/L) | 77.49±26.37 | 76.97±25.56 | 0.927 |
| GGT (U/L) | 42.57±47.01 | 39.38±48.33 | 0.296 |
| Albumin (g/L) | 44.51±5.51 | 44.42±4.72 | 0.438 |
| TBil (µmol/L) | 17.41±27.09 | 16.01±9.57 | 0.788 |
| PT (s) | 12.52±1.35 | 12.66±1.73 | 0.548 |
| PLT (×10^9^/L) | 171.57±54.73 | 173.90±67.28 | 0.991 |
| Hyaluronic (ug/L) | 115.13±72.52 | 115.50±68.54 | 0.719 |
| Laminin (ug/L) | 77.13±148.33 | 98.47±225.35 | 0.869 |
| PIIINP (ug/L) | 3.64±5.64 | 3.67±3.55 | 0.292 |
| Collagen IV (pg/mL) | 876.78±484.71 | 937.38±638.63 | 0.506 |
| YKL-40 (log_10_ pg/mL) | 4.47±0.38 | 4.45±0.36 | 0.456 |
| sCD163(log_10_ pg/mL) | 6.00±0.33 | 6.01±0.32 | 0.900 |
| MMP-1 (log_10_ pg/mL) | 3.48±0.31 | 3.49±0.32 | 0.629 |
| MMP-2 (log_10_ pg/mL) | 5.26±0.10 | 5.27±0.10 | 0.779 |
| MMP-3 (log_10_ pg/mL) | 4.18±0.27 | 4.16±0.24 | 0.386 |
| MMP-9 (log_10_ pg/mL) | 4.86±0.44 | 4.90±0.48 | 0.481 |
| TIMP-1 (log_10_ pg/mL) | 5.06±0.13 | 5.05±0.13 | 0.712 |

*BMI* body mass index, *HBsAg* hepatitis B surface antigen, *ALT* alanine transaminase, *AST* aspartate transaminase, *ALP* alkaline phosphatase, *GGT* gamma-glutamyl transpeptidase, *TBil* total bilirubin, *PT* prothrombin time, *PLT* platelet counts, *PIIINP* N-terminal peptide of type III procollagen, *YKL-40* chitinase 3-like-1, *sCD163* soluble CD163, *MMP* matrix metalloproteinase, *TIMP-1* tissue inhibitor of metalloproteinase 1. Data presented as mean ± SD or no. (%).

**Supplementary Table 3.** Comparisons of the five noninvasive fibrosis models.

|  | **Advantages** | **Limitations** | **Clinical situational uses** |
| --- | --- | --- | --- |
| YKL-40 Model | simple model (AST, PLT, Hyaluronic, YKL-40) ; modeling in CHB patients. | lacking of prospective cohort validation | CHB patients with ALT<2ULN; significant fibrosis (F≥3) (ISHAK) |
| APRI | simple model (AST, PLT) | modeling in CHC patients | significant fibrosis (F≥3) and cirrhosis (ISHAK) |
| FIB-4 | simple model (Age, ALT, AST, PLT) | modeling in CHC patients | severe fibrosis (F3-4) and cirrhosis (METAVIR index) |
| Forns’ index | simple model (PLT, GGT, Age, Cholesterol) | modeling in CHC patients | significant fibrosis (stages 2-4) (Scheuer’s classification) |
| Hui model | simple model (BMI, PLT, Albumin, TBil); modeling in CHB patients. | lacking of large clinical cohort validation (modeling in 235 patients) | significant fibrosis (F3-6) (ISHAK) |
